# Performance Comparison of Convolutional Neural Network-Based Hearing Loss Classification Model Using Auditory Brainstem Response Data

**DOI:** 10.3390/diagnostics14121232

**Published:** 2024-06-12

**Authors:** Jun Ma, Seong Jun Choi, Sungyeup Kim, Min Hong

**Affiliations:** 1 Department of Software Convergence, Soonchunhyang University, Asan 31538, Republic of Korea; ringring369@gmail.com; 2 Department of Otorhinolaryngology—Head and Neck Surgery, College of Medicine, Soonchunhyang University Cheonan Hospital, Cheonan 31151, Republic of Korea; akas9238@hanmail.net; 3 Insitute for Artificial Intelligence and Software, Soonchunhyang University, Asan 31538, Republic of Korea; sungyeup.kim@gmail.com; 4 Department of Computer Software Engineering, Soonchunhyang University, Asan 31538, Republic of Korea

**Keywords:** auditory brainstem response, ABR, deep learning, VGG16, VGG19, DenseNet121, Densenet201, Alexnet, image processing, hearing loss

## Abstract

This study evaluates the efficacy of several Convolutional Neural Network (CNN) models for the classification of hearing loss in patients using preprocessed auditory brainstem response (ABR) image data. Specifically, we employed six CNN architectures—VGG16, VGG19, DenseNet121, DenseNet-201, AlexNet, and InceptionV3—to differentiate between patients with hearing loss and those with normal hearing. A dataset comprising 7990 preprocessed ABR images was utilized to assess the performance and accuracy of these models. Each model was systematically tested to determine its capability to accurately classify hearing loss. A comparative analysis of the models focused on metrics of accuracy and computational efficiency. The results indicated that the AlexNet model exhibited superior performance, achieving an accuracy of 95.93%. The findings from this research suggest that deep learning models, particularly AlexNet in this instance, hold significant potential for automating the diagnosis of hearing loss using ABR graph data. Future work will aim to refine these models to enhance their diagnostic accuracy and efficiency, fostering their practical application in clinical settings.

## 1. Introduction

Convolutional Neural Networks (CNNs) are a specialized category of deep learning algorithms predominantly utilized in the fields of image and video recognition. Characteristically, CNNs automate the process of learning and classifying image features through a structured network comprising convolutional layers, pooling layers, and fully connected layers. The convolutional layer primarily serves to extract pertinent features from images, while the pooling layer reduces computational load by diminishing the spatial dimensions of the data. The fully connected layer then performs the final task of classification. These networks are extensively applied across various tasks in computer vision, including image classification, object detection, and face recognition, due to their robustness in handling complex visual inputs [1,2]. In the realm of medical imaging, CNNs assume a critical role given the intricate nature of most medical datasets. They provide effective mechanisms for processing and interpreting such data swiftly, which is indispensable in clinical settings. Consequently, CNNs are employed in diverse medical imaging applications encompassing disease classification, tissue categorization, and image segmentation, among others [3]. This versatility underlines the significance of CNNs in advancing medical image analysis and improving diagnostic methodologies.

The Auditory Brainstem Response (ABR) is an electrophysiological measurement reflecting the brainstem’s activity in response to auditory stimuli. This response involves the transmission of a neuroelectric signal from the cochlea through the auditory pathways to the auditory cortex of the brain. The ABR test, a diagnostic procedure used to assess hearing functionality, measures the waveform of this electrical response. This test is particularly valuable in clinical settings for evaluating hearing impairment. Its non-invasive nature and independence from patient consciousness—being unaffected by sleep or anesthesia—make it particularly suitable for use in populations unable to provide reliable auditory feedback. These include newborns, infants, young children, the elderly, and individuals with congenital disabilities. Consequently, the ABR test provides a robust and objective method for assessing auditory function across a diverse patient demographic [4].

ABR measurement is a neurophysiological method used to record changes in brain waves triggered by auditory stimulation. This technique involves the application of click sounds at intervals of approximately 0.8 ms combined with energy modulation during auditory transmission to stimulate brain wave activity. When auditory stimuli ranging from 10 dB to 100 dB are administered, typically, five to seven waves are detectable. In normal adults, a waveform responsive to the stimulus typically emerges within approximately 10 milliseconds following the onset of the click sound [5]. Among the identifiable waves, wave number 5 (V wave) is particularly significant for clinical assessments. The threshold of hearing is determined by analyzing the latency periods of the V wave across various dB levels. Hearing loss is subsequently diagnosed based on these latency values within the specified dB range [6].

**Figure 1:**
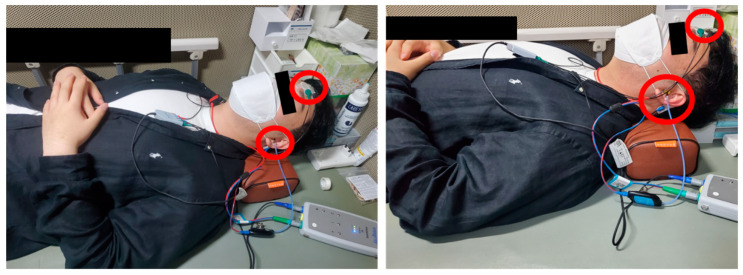
Auditory brainstem response test scene.

During the ABR testing procedure, as illustrated in Figure 1, small electrodes marked by red circles are affixed to the subject’s forehead and behind the ears. These electrodes detect electrical activity within the auditory nerve and brainstem in response to auditory stimuli. The test involves the administration of a series of click sounds delivered through an eartip inserted into the ear, with the brain’s responses—essentially, brain waves—being detected and automatically recorded by a computer system. This method offers an objective assessment of hearing functionality, contrasting with other methods that rely on subjective patient responses. In the process of measurement, the audiologist identifies and records the V wave, which is critical for hearing and occurs between 6 ms and 8 ms, from among wave information labeled from 1 to 5, corresponding to each decibel (dB) stimulus level. The measurement concludes once the waveforms for all dB stimulus levels have been successfully recorded [7].

**Figure 2:**
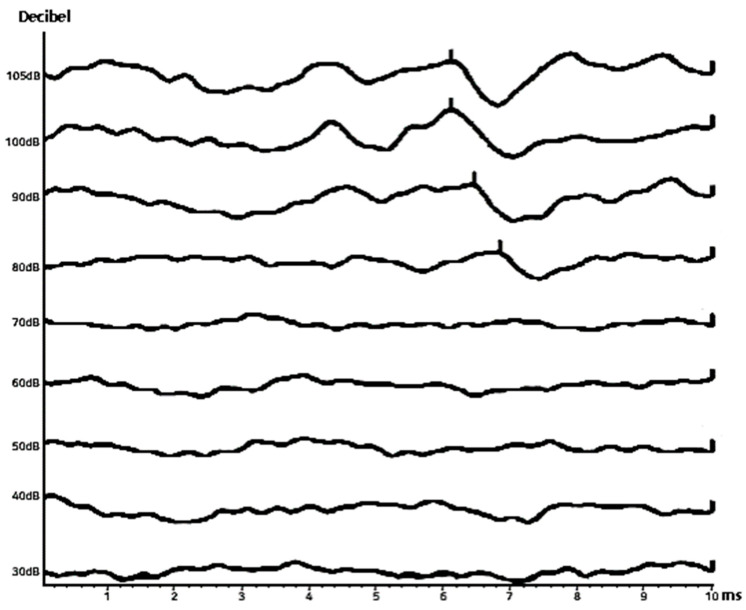
Auditory brainstem response data after pre-processing.

In our previous study [8], we conducted preprocessing to standardize the auditory brainstem response (ABR) graph outputs across various manufacturers. ABR graph data from five different manufacturers—Audera, Navigator, Eclipse, Viking Select, and Interacoustics—were collected. Each ABR graph was normalized as depicted in Figure 2, resulting in a dataset comprising 10,000 data entries. Furthermore, during the preprocessing phase, a total of 2010 images were filtered out due to significant reductions in graph resolution or improper graph outputs. Consequently, the analysis was conducted on 7990 images using normalized ABR data from the remaining valid datasets.

## 2. Materials and Methods

### 2.1. Auditory Brainstem Response Data

The ABR graph serves as a crucial diagnostic tool for evaluating the auditory system by visualizing changes in electrical activity over time. An analysis of the ABR graph can reveal significant insights into the auditory system’s condition through various characteristic features described below.

Multi-wave form: the ABR graph typically displays a series of waves, each sequentially representing electrical activity in different parts of the brain at specific times.

The size and spacing of waves: the initial wave usually appears as the largest and most distinct wave, with subsequent waves diminishing progressively in size and becoming closer together.

Latency and amplitude: these parameters are critical; latency refers to the timing of the wave’s occurrence post stimulus, and amplitude denotes the wave’s magnitude.

Baseline: the baseline of the graph indicates normal brain activity levels; any deviation from this baseline may suggest abnormalities in the auditory pathway.

Axes: the ABR graph is typically oriented with time (ms) on the horizontal axis and amplitude (µV) on the vertical axis, where sound pressure levels (decibels, dB) may also be considered.

**Figure 3:**
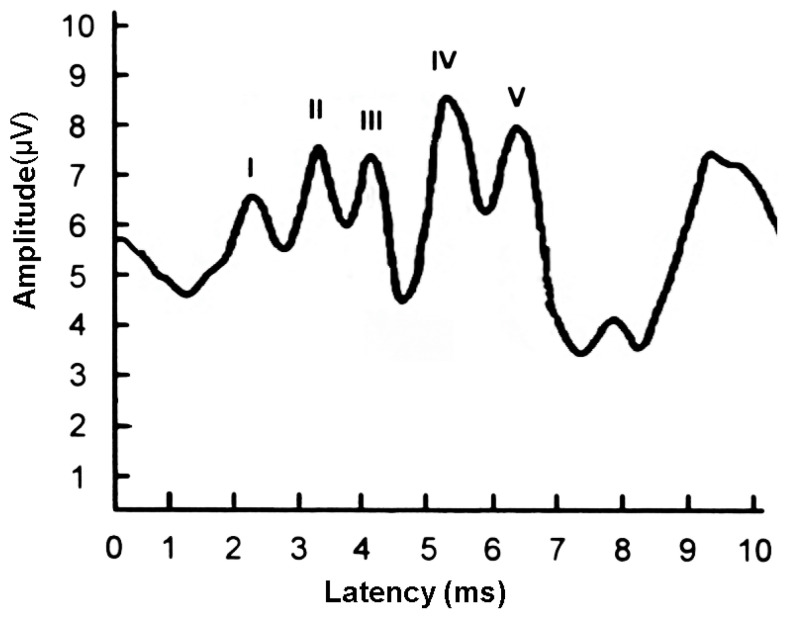
Auditory brainstem response example graph.

By systematically assessing these features—particularly changes in latency and amplitude—anomalies such as hearing impairment or disorders within the central auditory pathway can be detected. Thus, the ABR graph not only aids in assessing the patient’s hearing status but also contributes to the formulation of an appropriate treatment plan [9,10,11,12]. The methodology for analyzing an ABR graph, as depicted in Figure 3, is essential for comprehensively evaluating both hearing function and the broader state of the auditory system.

Wave I: This initial wave is indicative of the acoustic signal’s arrival at the auditory nerve. The latency for Wave I, representing the time taken for the stimulus to reach the auditory nerve, typically ranges from 1 to 4 ms.

Wave II: occurring in the auditory brainstem, specifically in the region associated with the auditory pathway’s initial processing stages, the latency from Wave I to Wave II measures the transmission time to the auditory brainstem and is generally observed between 2 and 4 ms.

Wave III: this wave is generated in the cochlear nuclei—the Dorsal Cochlear Nucleus (DCN) and Ventral Cochlear Nucleus (VCN)—located in the lower auditory brainstem. The latency from Wave II to Wave III, which measures the passage of stimulus through the auditory brainstem, typically ranges between 3 and 5 ms.

Wave IV: Representing signals generated en route to the Medial Superior Olive (MSO) at the upper part of the auditory brainstem; the latency between Wave III and Wave IV usually spans 4 to 5.5 ms.

Wave V: The largest of the acoustic signals, Wave V emanates from the output region of the auditory brainstem, reaching the Inferior Colliculus (IC). The latency from Wave IV to Wave V is noted between 5.5 and 7 ms.

Wave latency: the latency of each wave quantifies the time required for its generation. Within a normal auditory system, these latencies fall within specific ranges; however, abnormalities may manifest as delayed latencies.

Wave amplitude: The amplitude of each wave reflects its magnitude. Typically, a healthy auditory system produces waves of a large and consistent amplitude. Reduced amplitude may indicate auditory abnormalities.

Interpeak latency: This metric illustrates the latency differences between consecutive waves, reflecting the conduction time along the central auditory pathway. Normal auditory systems exhibit consistent interpeak latencies, whereas increased values may suggest central auditory pathway dysfunction.

These metrics provide a comprehensive framework for assessing the integrity and functionality of the auditory system through ABR testing, facilitating the identification and characterization of potential auditory impairments.

**Figure 4:**
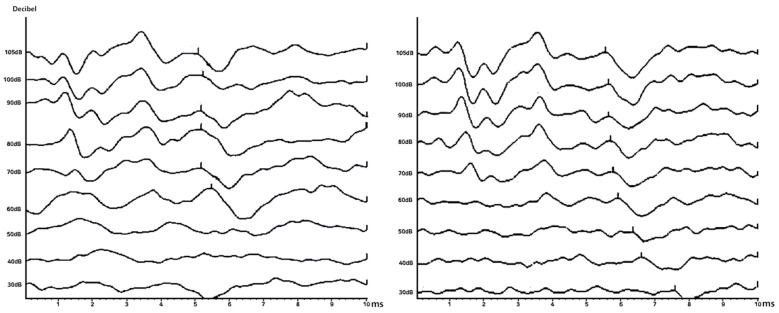
An ABR graph of a patient with hearing loss (**left**) and an ABR graph of a normal person (**right**).

Hearing loss: Hearing loss is characterized as a reduction in the ability to perceive or interpret sounds; the loss is attributable to anomalies within the auditory system, which may involve the external or internal ear structures or the auditory nerve. This condition can be either temporary or permanent and may affect one or both ears. Within the context of this study, the severity of hearing loss is assessed based on the detection of the V wave in the ABR graph data, as illustrated in Figure 4. Typically, V waves are elicited by sound stimuli ranging from 10 to 100 dB, presented in 10 dB increments. An absence of V waves in waveforms at or below 40 dB typically leads otolaryngologists to diagnose hearing loss.

The analysis of an ABR graph involves a detailed evaluation of various parameters to ascertain the auditory system’s status. Key indicators include the latency and amplitude of waves; a delay in wave latency or a reduction in amplitude may signal auditory impairments or issues within the central auditory pathway. By examining these attributes, clinicians can effectively gauge a patient’s hearing condition and devise appropriate treatment strategies. Such diagnostic practices are crucial for the early detection and management of hearing loss, thereby enhancing the quality of life and communication abilities of affected individuals [13,14,15,16].

### 2.2. CNN Classification Model

In our previous study, we evaluated the classification of hearing loss using solely the VGG16 model [8]. Expanding upon this initial approach, the current study incorporates a broader array of convolutional neural network models, specifically VGG16, VGG19, DenseNet121, DenseNet201, AlexNet, and InceptionV3, to perform more comprehensive learning and classification tests. Each model, recognized for its unique strengths and limitations, has previously been utilized across a variety of medical image classification tasks. For the purposes of this study, we tailored the hyperparameters, specifically the batch size and layer configurations, to optimize the learning process for ABR image classification. This part details the architectural nuances and characteristics of each model and describes the specific modifications made to the hyperparameters to enhance model performance for this application. These adjustments are pivotal in refining our approach to accurately classifying hearing loss through deep learning techniques.

#### 2.2.1. VGG16 and VGG19

The VGG model, developed by the Visual Geometry Group at Oxford, includes two primary configurations: VGG16 and VGG19. VGG19 extends the architecture of VGG16 by adding three additional convolutional layers positioned before the 3rd, 4th, and 5th max pooling layers, enhancing its depth and complexity. In our experiments with the VGG16 model, the original images, sized 573 × 505 pixels, were initially resized to 224 × 224 pixels. Subsequently, the images were further scaled down to dimensions of 286 × 252, 143 × 126, 71 × 63, 35 × 31, and 17 × 15 to facilitate object recognition. The learning process involved adjustments in the dense layer configurations, with neuron counts set to 1024, 512, and 2, optimizing the network’s ability to discern features at various scales. The VGG19 model, with its additional convolutional layers, retains the number of layers for sizes 286 × 252 and 143 × 126 but adds a layer each at smaller scales (71 × 63 and smaller), comprising a total of 19 layers to enhance detail recognition [17,18,19,20,21]. In a related study conducted by Dey et al., a pneumonia detection model utilizing VGG19 applied to chest X-ray images demonstrated a high classification accuracy of up to 97.94% [22]. Similarly, Mateen et al. reported that the VGG19 model was effectively utilized in medical image analysis, achieving an impressive classification accuracy of 98.13% in a retinopathy classification system using fundus images [23]. For the purpose of this study, which was tailored to ABR image classification, the VGG19 model and the VGG16 model were enhanced by incorporating two additional dense layers and a dropout layer in each configuration to prevent overfitting. The learning framework was structured with dense layers of 1024, 512, 256, 128, and 2 neurons, with a batch size of 8, serving as a robust hyperparameter setup. This architecture was designed to maximize the model’s ability to accurately classify ABR images, leveraging deeper layers for more nuanced feature extraction.

#### 2.2.2. DenseNet121 and DenseNet201

DenseNet is a CNN model engineered to enhance training efficiency by integrating the concept of shorter connections. This design enables direct links between the input and output layers, fostering a deeper and structurally more efficient network capable of delivering precise performance outcomes. Unlike traditional CNNs, which feature connections primarily to the immediately subsequent layer, DenseNet boasts a comprehensive connection structure with L(L + 1)/2 direct connections, greatly enriching the flow of information across the network. To efficiently manage down-sampling, the architecture is segmented into three distinct dense blocks. Each block is separated by a transition layer which performs both convolution and pooling operations, thus maintaining the network’s depth while progressively reducing its dimensionality [24,25,26,27,28]. In a related study by Chauhan et al., a DenseNet model was employed to differentiate COVID-19 patients from healthy individuals using chest X-ray images, achieving an impressive accuracy rate of 98.45% [29]. Within the context of this paper, the DenseNet121 and DenseNet201 models, comprising 121 and 201 layers, respectively, were utilized. Tailored specifically for ABR image classification, the learning process was conducted using dense layers configured with 256 and 2 neurons and a hyperparameter setting of a batch size of 8. This configuration was designed to optimize the network’s capability for high-accuracy classification in ABR imaging.

#### 2.2.3. AlexNet

AlexNet, a CNN model, significantly impacted the field of deep learning after securing victory in the 2012 ImageNet Large Scale Visual Recognition Challenge (ILSVRC). Named after Alex Krizhevsky, the lead author of the seminal paper “ImageNet Classification with Deep Convolutional Neural Networks”, AlexNet’s architecture has been instrumental in advancing CNN development. Its structure features a sequential layout comprising an input layer and five convolutional layers—each accompanied by a max pooling layer and a normalization layer—culminating in a dense layer dedicated to classification tasks [30,31,32,33]. In research conducted by Chen et al., the efficacy of various models including 3DAlexNet, ResNet50, and InceptionV4 was evaluated for the classification of magnetic resonance images to diagnose prostate cancer, yielding classification accuracies of 92.1%, 87.6%, and 85.7%, respectively [34]. Another study by Titoriya and Sachdeva utilized the AlexNet model to classify breast cancer tissue images, demonstrating a high classification accuracy of 95.7%, thereby underscoring its potential for medical imaging applications [35]. In this study, modifications were made to the original AlexNet architecture to enhance its suitability for Auditory Brainstem Response (ABR) image classification. Adjustments included the integration of max pooling layers at the first and fifth convolutional layers and the insertion of dropout layers within each dense layer to mitigate overfitting. The learning process was optimized by setting the hyperparameters of the dense layers to 4096, 4096, and 2, with a batch size of 8, facilitating an improved learning rates and robust classification performance in ABR image analysis.

#### 2.2.4. InceptionV3

Generally, there is a correlation between increased model size and both accuracy and computational effort. For instance, the DenseNet architecture enhances performance by deepening the model with skip connections, yet this also escalates computational demands, resulting in longer training durations due to the increased depth. Similarly, enlarging model size augments computational requirements, which presents a limitation when operating within memory constraints. The Inception model, devised by Google, addresses this challenge by employing convolutional decomposition to expand the model size while minimizing computational costs. The InceptionV3 model, which was utilized in this research, stands out among the Inception series with its 42-layer deep network, which is optimized to maintain a balance between a low parameter count and computational efficiency, akin to that of the VGG models [36,37]. In research conducted by Wang, Cheng, et al., the InceptionV3 model was applied to develop a classification system for lung nodules using chest X-ray images, achieving a classification accuracy of up to 86.4% [38]. In the context of this study, the InceptionV3 model was adapted for ABR image classification. Modifications were made to the model’s configuration, setting the hyperparameters of the dense layers to 256 and 2, and the batch size to 8, to tailor the learning process specifically for ABR image analysis. This strategic adjustment aims to leverage the model’s efficiency and deep learning capabilities for precise ABR image classification.

## 3. Results

### Model Training and ABR Data Classification Results

Using 7990 ABR data excluding impure data, learning and classification tests were conducted with 4794, 1598, and 1598 train, validation, and test data at a ratio of 6:2:2, respectively. The accuracy, loss results, and test classification confusion matrix results of each model’s learning are as follows.

**Figure 5:**
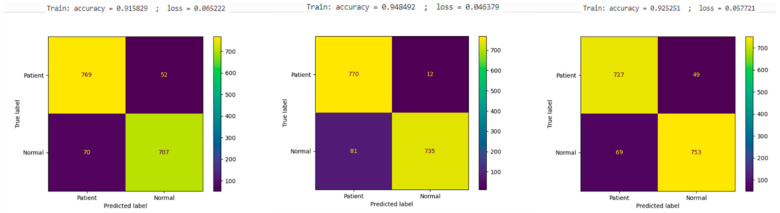
Learning results of VGG16 model (**left**), VGG19 model (**middle**), and DenseNet121 model (**right**).

The results in Figure 5 highlight the performance metrics for the VGG16 model during training, showing an accuracy of 91.58% and a loss of 6.52%. The confusion matrix for the test dataset for this model indicated 769 true negatives (tn), 52 false negatives (fn), 707 true positives (tp), and 70 false positives (fp). The VGG19 model demonstrated improved training performance, achieving an accuracy of 94.84% and a loss of 4.64%. The test data for this model produced a confusion matrix with 770 tn, 12 fn, 735 tp, and 81 fp. Additionally, the DenseNet121 model recorded a training accuracy of 92.52% and a loss of 5.77%, with its confusion matrix displaying 727 tn, 49 fn, 753 tp, and 69 fp.

**Figure 6:**
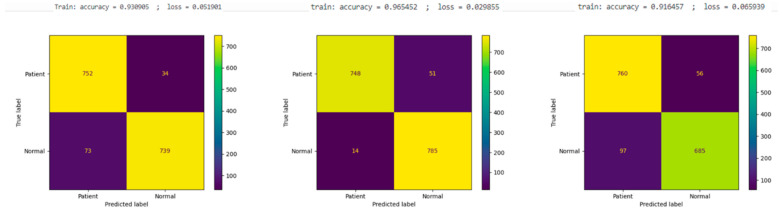
Learning results of DenseNet201 model (**left**), AlexNet model (**middle**), and InceptionV3 model (**right**).

And the results in Figure 6, the DenseNet201 model’s training performance exhibited an accuracy of 93.09% and a loss of 5.19%, with the confusion matrix for the test dataset indicating 752 tn, 34 fn, 739 tp, and 73 fp. The AlexNet model, on the other hand, achieved a training accuracy of 96.54% and a remarkably lower loss of 2.99%. The corresponding confusion matrix demonstrated its high precision with 748 tn, 51 fn, 785 tp, and only 14 fp, underscoring its efficacy in accurately classifying the conditions with minimal misclassifications. Lastly, the InceptionV3 model registered a training accuracy of 91.64% and a loss of 6.59%, with its test data confusion matrix revealing 760 tn, 56 fn, 685 tp, and 97 fp.

Based on the confusion matrix results from the test data for each model, various performance metrics were calculated and systematically tabulated. These metrics include accuracy, the true negative rate (TNR), the true positive rate (TPR), the false positive rate (FPR), the false negative rate (FNR), precision, and the F1 score. The formulas for each of these metrics are outlined below, with their respective results being derived from Equations (1)–(7):

(1) $$\mathrm{Accuracy}=\frac{tp+tn}{tp+tn+fp+fn}$$

Accuracy: this is calculated as the ratio of correctly predicted observations (both true positives and true negatives) to the total observations in the dataset.

(2) $$\mathrm{TNR}=\frac{tn}{tn+fp}$$

True negative rate (TNR), also known as specificity: this measures the proportion of actual negatives that are correctly identified.

(3) $$\mathrm{TPR}=\frac{tp}{tp+fn}$$

True positive rate (TPR), also known as sensitivity or recall: this metric indicates the proportion of actual positives that are correctly identified.

(4) $$\mathrm{FPR}=\frac{fp}{fp+tn}$$

False positive rate (FPR): this is calculated as the ratio of the number of false positives to the sum of the false positives and true negatives.

(5) $$\mathrm{FNR}=\frac{fn}{fn+tp}$$

False negative rate (FNR): this measures the proportion of positives which yield negative test outcomes with the model.

(6) $$\mathrm{Precision}=\frac{tp}{tp+fp}$$

Precision is also known as the positive predictive value: this is the ratio of true positives to the combined total of true positives and false positives.

(7) $$F1\;\mathrm{score}=2\times \frac{precision\times TPR}{precision+TPR}$$

F1 score: this is the harmonic mean of Precision and Recall, providing a balance between the two when their rates may vary.

The calculated values for these metrics are compiled in a Table 1 within this paper, providing a comprehensive assessment of each model’s performance on the test dataset. This structured approach allows for a detailed comparison and evaluation of the effectiveness of each classification model in the context of hearing loss detection.

**Figure 7:**
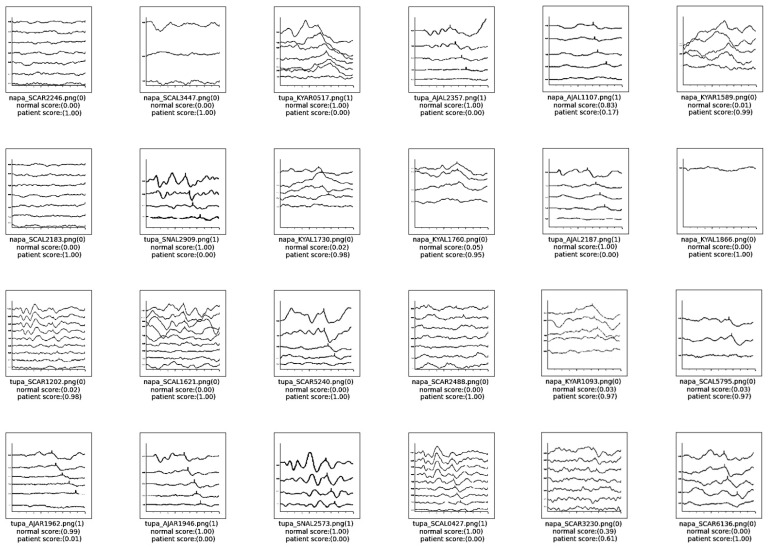
Classification result scores of AlexNet model.

The result of the classification models utilized in this research were evaluated based on outputs of classification scores. Figure 7 presents the classification score results for the AlexNet model, which demonstrated the highest accuracy among the models tested. This figure displays image data that was randomly selected from the test dataset. It includes the filename of the data—where “napa” denotes an image associated with hearing loss and “tupa” indicates a normal hearing image. Additionally, the outcomes of the classification process are indicated, with the number 0 representing hearing loss and the number 1 representing normal hearing. The scores leading up to these classifications are also documented to provide a comprehensive view of the model’s performance in distinguishing between the two categories. This detailed display of results facilitates an understanding of the model’s efficacy in accurately classifying auditory conditions based on ABR image data.

These findings illustrate the varying levels of performance and accuracy across the models tested, offering insights into their respective strengths and areas for improvement in the classification of conditions based on the training and validation datasets.

## 4. Discussion

### 4.1. Classification Data Analysis

In the current study, the classification learning revealed that AlexNet achieved the highest overall accuracy, recording 95.93%. However, when focusing specifically on the accuracy of hearing loss classification, as measured by the TNR, VGG19 excelled with a TNR of 98.47%, making it the most effective model for this particular objective. Given that the primary aim of this research is to accurately classify hearing loss, the VGG19 model emerges as the superior performer in this context. Nevertheless, it is important to consider the structural differences between the models. AlexNet, with its comparatively shallower layer depth, consumes fewer temporal resources during the learning classification process. Thus, for applications involving the classification of ABR data on a scale larger than the current dataset of 7990 cases, AlexNet presents a viable option due to its efficiency in handling larger datasets without a significant increase in computational demand. This balance between accuracy and efficiency is crucial for scaling the application of these models to larger datasets in future studies.

### 4.2. Analysis of ABR Data That Are Not Classified Correctly

In this part, we will discuss the results that were not classified correctly among the classification results from various models.

#### 4.2.1. False Negative: In Case the Data Are actually Normal but Are Classified as a Patient with Hearing Loss

**Figure 8:**
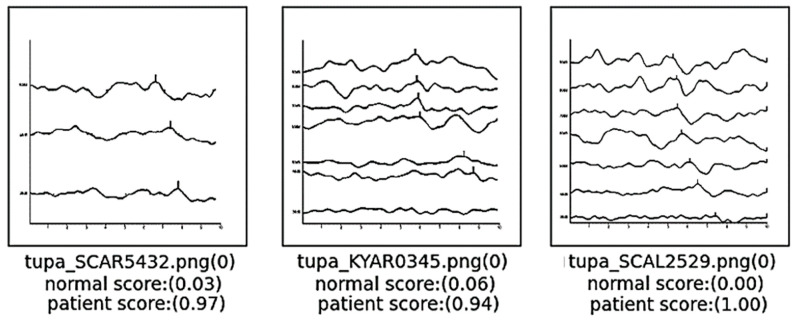
Cases of false negatives.

Figure 8 presents a selection of misclassified cases identified through the application of the AlexNet, VGG19, and VGG16 models within the classification analyses of this study. These instances involve subjects who, despite having normal hearing, were erroneously classified as suffering from hearing loss. The graph on the left represents ABR data from a 1-year-old infant, with V waves detected at 60 dB, 40 dB, and 30 dB. The analysis suggests that the model’s misclassification may stem from the limited number of data points in this sample, contrasting with the more comprehensive ABR data typically gathered from the general population, which is measured in 10 dB increments from 30 dB to 90 dB. The middle graph displays ABR results for a 52-year-old individual; no V wave was detected at 30 dB. This subject, diagnosed with normal hearing by a medical professional, was analyzed in comparison to typical public ABR data, which usually exhibits V waves across all tested decibels. The absence of a V wave at 30 dB in this case led to a model misrecognition, highlighting a deviation from expected patterns observed in broader datasets. The graph on the right documents ABR measurements for an 18-year-old individual. The analysis determined that the model misclassification occurred because the final 30 dB graph plotted very close to the x-axis. This proximity likely influenced the model’s perception, causing it to misidentify the presence of a V wave, which deviates from the normative data where V waves are consistently present across all measurements. These illustrations underscore challenges with model accuracy when faced with atypical data representations, emphasizing the need for the continuous refinement of classification algorithms to enhance diagnostic precision in clinical settings.

#### 4.2.2. False Positive: In Case the Data Represent Patients with Actual Hearing Loss but Classified as Normal

**Figure 9:**
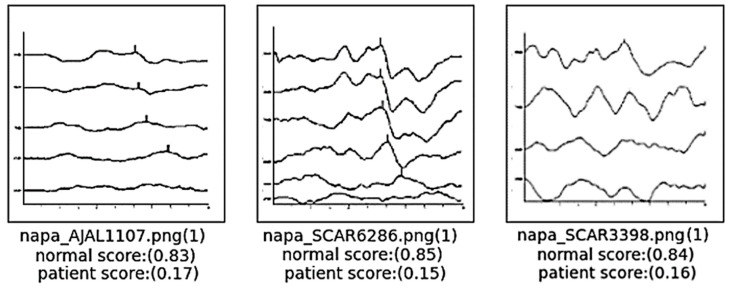
Cases of false positives.

Figure 9 presents images indicative of hearing loss that were inaccurately classified as normal by the model. For the images on the left and right, prior to the existing preprocessing steps, the resolution was significantly compromised, necessitating further preprocessing to enhance resolution quality. Despite these efforts, the resolution remained comparatively lower than that of typical ABR graphs, which likely impeded the model’s ability to accurately classify these cases. Concerning the image in the middle, the analysis suggests that the misclassification occurred due to the proximity of the wave in the bottom graph to the x-axis and its closeness to the graph directly above it. This spatial arrangement may have confused the model, leading to an incorrect interpretation of the data. This observation underscores the sensitivity of classification models to variations in graphical representation and highlights the need for robust preprocessing techniques to ensure consistent image quality across all data inputs. Such improvements are critical for enhancing the accuracy of diagnostic models in clinical applications.

## 5. Conclusions

In this study, we developed multiple models to classify hearing loss using preprocessed ABR graph data and evaluated their comparative performances. The AlexNet model exhibited the highest accuracy with a value of 95.93%, while the VGG19 model demonstrated the best TNR at 98.47%. Among the six evaluated models, AlexNet showed the quickest learning speed, followed by InceptionV3, VGG16, VGG19, DenseNet121, and DenseNet201 in terms of processing time. For instances in which images are incorrectly classified, future work will involve exploring further supplementation and preprocessing strategies. These will aim to enhance image quality without compromising the integrity of the original data including measures such as increasing resolution, augmenting the X and Y axes, and adjusting the wave positioning for each decibel level.

Previously, the process of diagnosing hearing loss using ABR involved audiologists and otolaryngologists manually reviewing each ABR graph, which was time-consuming. This study was conducted to address this issue and improve the efficiency of the diagnostic process. The findings of this research pave the way for the development of a robust model that can support the preliminary automatic classification of ABR data, assisting in the pre-diagnostic stages before clinical evaluation by a physician. Additionally, the study plans to extend into the creation of an automatic V-latency detection algorithm which will be designed for universal application across various devices rather than being confined to specific equipment. This advancement is anticipated to simplify the diagnosis of hearing loss and related auditory conditions, thereby enhancing patient care and diagnostic efficiency.

## Figures and Tables

**Table 1:** Total data validity calculation results.

	Accuracy	TNR	TPR	FPR	FNR	Precision	F1 Score
VGG16	92.37%	93.67%	90.99%	6.33%	9.01%	93.15%	0.9206
VGG19	94.18%	98.47%	90.07%	1.53%	9.93%	98.39%	0.9405
DenseNet121	92.62%	93.69%	91.61%	6.31%	8.39%	93.89%	0.9273
DenseNet201	93.30%	95.67%	91.01%	4.33%	8.99%	95.60%	0.9325
AlexNet	95.93%	93.62%	98.25%	6.38%	1.75%	93.90%	0.9602
InceptionV3	90.43%	93.14%	87.60%	6.86%	12.40%	92.44%	0.8995

## Data Availability

Data sharing is not applicable.
